# Endodontic Treatment of a Maxillary Second Molar with Developmental Anomaly: A Case Report

**Published:** 2007-07-05

**Authors:** Saeed Asgary

**Affiliations:** 1 *Department of Endodontics, Iranian Center for Endodontic Research, Dental School, Shahid Beheshti University of Medical Sciences, Tehran, Iran*

**Keywords:** Case Report, Double Tooth, Endodontic, Fusion

## Abstract

Fusion is a rare occurrence in molar teeth. The purpose of this rare case presentation is to describe the nonsurgical endodontic treatment of maxillary molar. A 28-year-old patient was referred for endodontic treatment of her chronic apical abscess of right maxillary second molar. In the clinical examination, a sinus tract adjacent to involved tooth and a small crown of supernumerary tooth fused to the buccal surface of the molar at gingival margin was observed. Endodontic treatment was decided for the involved molar for functional reason. Recall examination, a year after completion of endodontic and restorative treatments, showed the tooth was clinically asymptomatic and there was no radiographic lucency around the apical region.

## INTRODUCTION

Dental fusion is defined as the merging of two or more teeth at enamel and/or dentinal level (1-2). The etiology of dental fusion is still unclear. One reason may be the influence of pressure or physical forces providing close contact between two adjacent tooth germs and thus resulting in the enamel organ and the dental papilla to unite (3).

Local metabolic interferences, which occur during differentiation of the tooth germ, may be another cause for dental fusion (4). Genetic determination may also be evident in some of the cases presented in the literature (5). In addition, other types of dental anomalies have been described (2,6):

a) Dehiscence: laceration resulting from trauma and affecting the crown of a tooth germ;

b) Concrescence: the merging of two or more teeth at root cementum;

c) Gemination (twinning): attempted division of a tooth germ in two;

d) Schizodontia: complete division of a tooth germ in two; and

e) Dens in dente: enamel penetration into the pulp chamber.

Dental fusion occurring particularly in anterior teeth with apparent equal distribution between the two jaws, and is more common during the deciduous dentition phase. It is very rare in molars. The prevalence of this dental anomaly is estimated at 0.1-2.5% of individuals (7-9).

Fused teeth may present two independent endodontic systems, one pulp chamber dividing into two root canals or, less often, a single root and one or two pulp chambers (1,10).

The fusion may be partial or total depending on the stage of tooth development at the time of union, including only the tooth crowns or the tooth crowns and roots, respectively (11). Although the most common situation is the fusion of one supernumerary with a normal tooth, the fusion of two normal teeth may occur, thus reducing the number of teeth in the arch (12);the fused tooth may be of normal size or larger than normal (13).

Dental fusion is generally asymptomatic and dose not requires any treatment. However, there could be poor aesthetics, periodontal damage or caries leading to pulp necrosis (4).

This case report describes the endodontic management of a maxillary second molar fused with a supernumerary.

**Figure 1:**
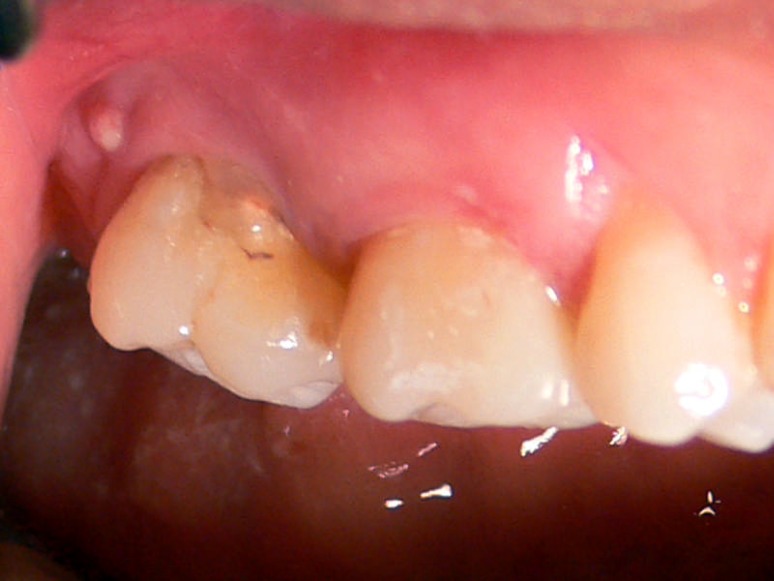
Intraoral view of the fused second maxillary molar and a parulis

**Figure 2:**
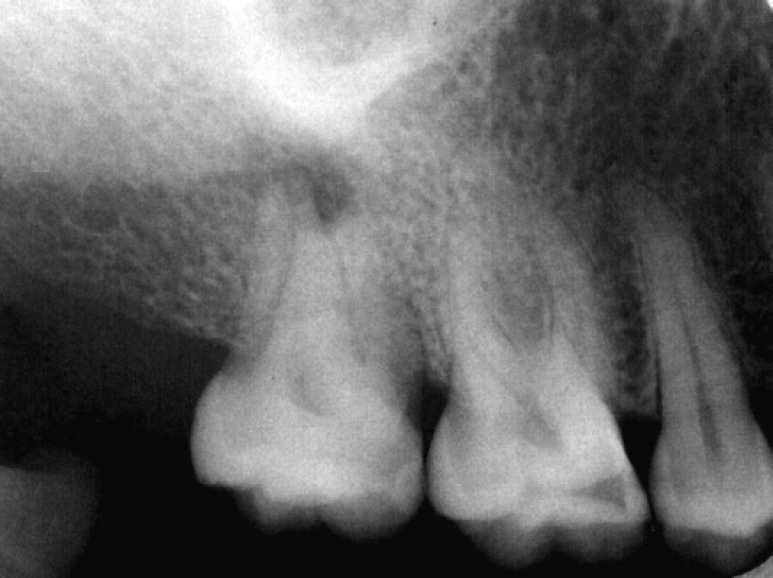
Preoperative radiograph demonstrated periapical radiolucency and abnormal roots

## CASE REPORT

A 28-year-old woman was referred for endodontic treatment of an anomalous tooth (maxillary right second molar). The patient complained of draining sinus tract in the gingiva of involved tooth (Figure 1),(Figure 2). Her medical history was noncontributory.

Clinical examination revealed that the maxillary right second molar was fused with a supernumerary. There was an extra small crown of abnormal appearance adjacent to the normal tooth. A draining sinus tract was present at the distobuccal of the fused tooth. No caries could be detected. The tooth displayed physiological mobility, but was sensitive to percussion. Thermal and electrical pulp testing didn’t give a normal response while probing revealed no periodontal pocketing around the tooth.

**Figure 3:**
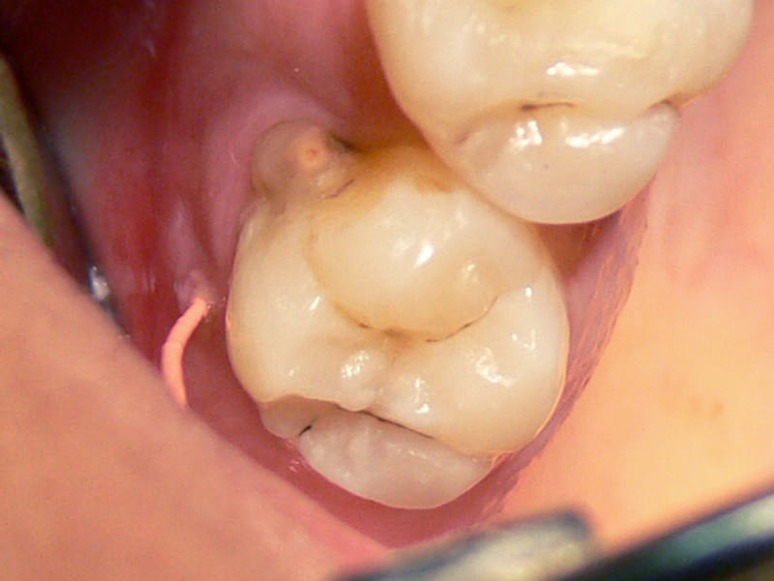
Occlusal view of fused teeth and traced gutta-percha placed in the sinus tract

**Figure 4:**
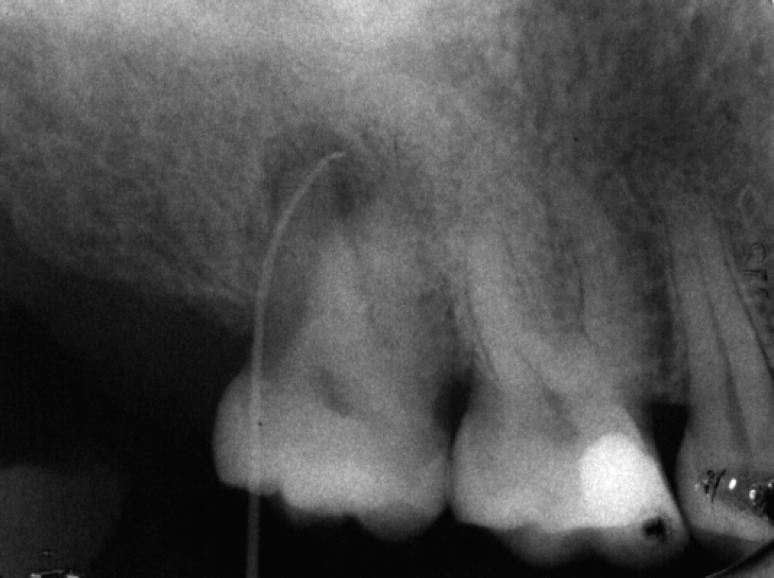
Tracing radiograph shows the origin of lesion is endodontic

Radiographic examination demonstrated mesio- buccal and distobuccal roots fused. A radiolucent area, 5 mm in diameter, with distinct borders was seen around the roots. Radiographic tracing with a gutta-percha cone revealed that the sinus tract led to the radiolucent area (Figure 3),(Figure 4). A diagnosis of necrotic pulp with chronic apical abscess of endodontic origin was established.

**Figure 5:**
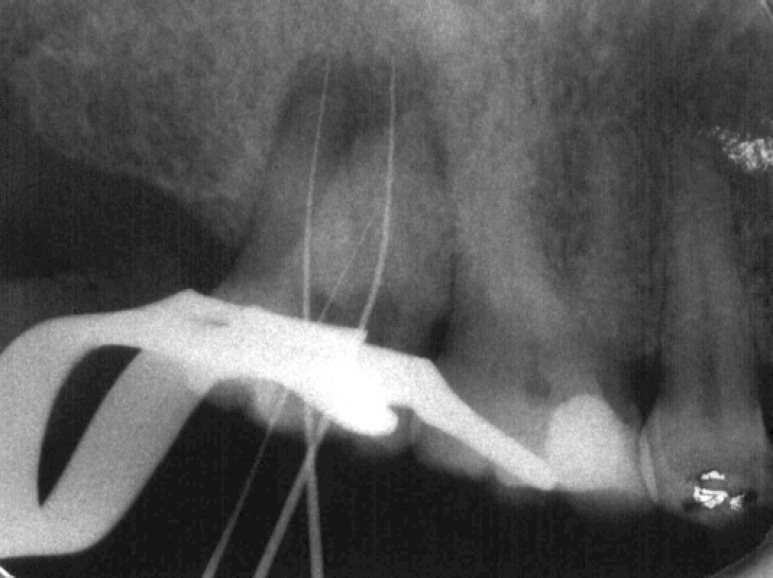
Working length determination X-ray

**Figure 6:**
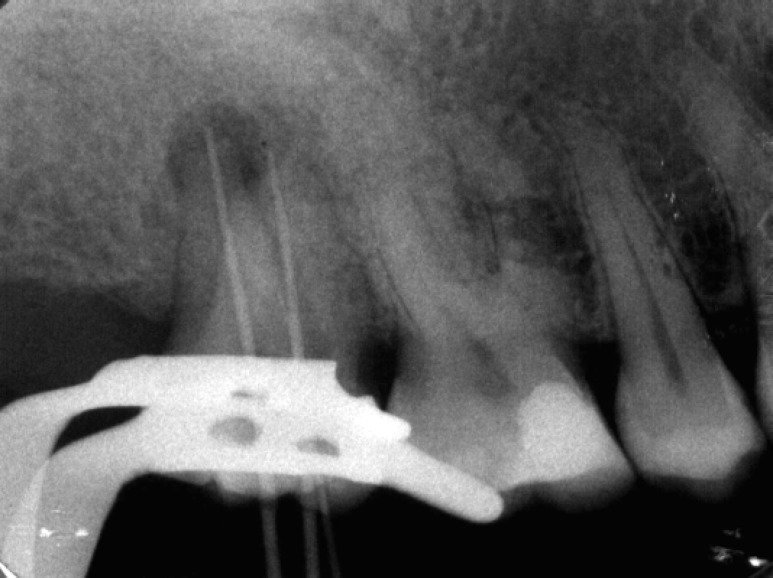
Master cone confirmation X-ray

**Figure 7:**
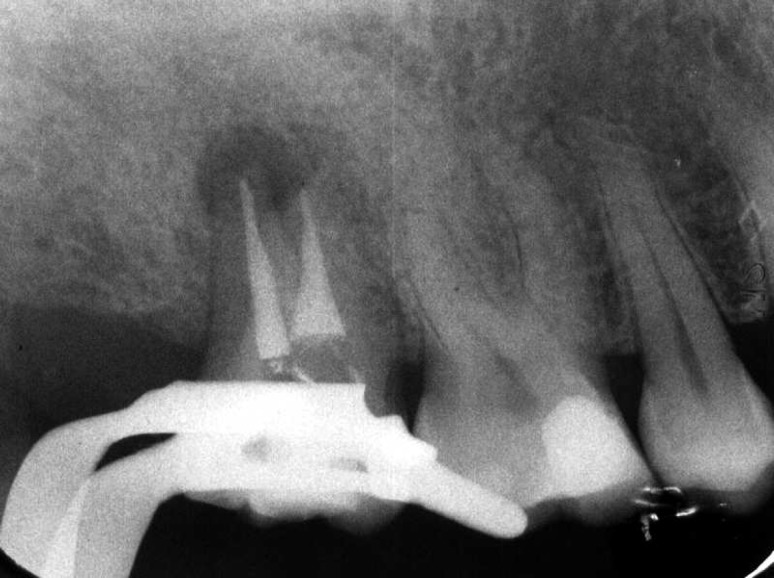
Postobturation radiograph

After local anesthesia (Lidocaine, 1:80000 epinephrine, Darupakhsh, Iran) and rubber dam isolation, working lengths were determined by radiograph (Figure 5) and chemomechanical preparation performed using K-files (Maillefer, LD Caulk Co., Milford, DE) with 2.5% sodium hypochlorite (Golrang, Tehran, Iran) solution as irrigant. After master cone confirmation (Figure 6), root canals were obturated using the lateral condensation technique with gutta-percha (Ariadent, Tehran, Iran) and Roth 801 root canal sealer (Roth Int. LTD, USA), and the coronal access sealed with IRM (Caulk, Milford, DE, USA) (Figure 7). A postoperative radiograph revealed densely condensed root canal fillings in the two canals. The crown was then restored permanently with amalgam.

**Figure 8:**
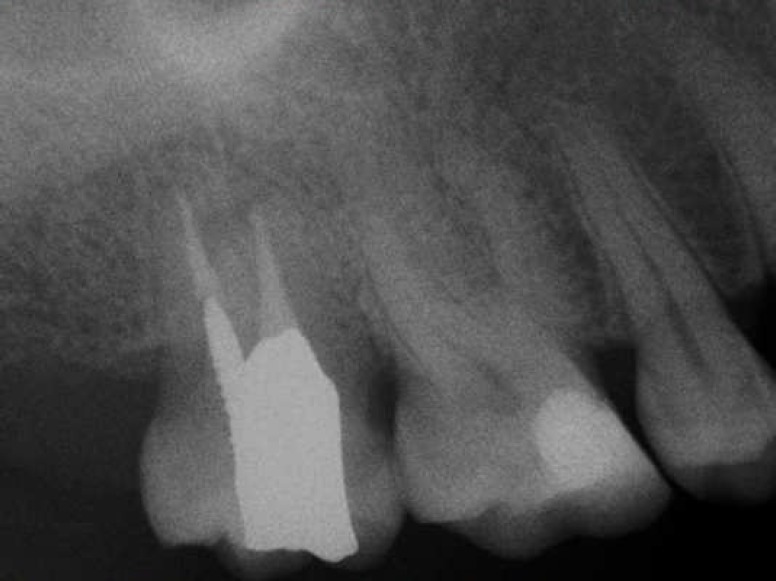
Recall radiograph revealed complete healing of periapical lesion

The recall examination after 1 year revealed that periodontal condition was healthy, the tooth was asymptomatic, and complete bone regeneration was seen on radiograph (Figure 8).

## DISCUSSION

As a common opinion, the clinical differentiation between dental fusion and germination is difficult. For the purpose of having better differentiation between these anomalies, it has been suggested that the teeth in the arch be counted with the anomalous crown counted as one. The presence of all teeth indicates gemination, whilst one tooth less than normal indicates fusion (14). Unfortunately, this rule is compromised when dental fusion or germination associated to dental agenesis or supernumerary teeth (15). As an illustrative example of such difficulty, a geminated second molar can be observed with agenesis of the wisdom tooth. However, there is no clinical importance in differential diagnosis of dental fusion and germination (16); many authors prefer to use the term “double tooth” or “fused teeth” in view of the uncertainty regarding the embryological cause underlying the junction defect or teeth joined together by dentine, respectively (8,17).

In this case, the history of wisdom tooth extraction, clinical observation of a small abnormal fused crown of a supernumerary tooth, and normal number of remaining teeth led us to established diagnosis of either dental fusion of the second maxillary molar with a supernumerary tooth or gemination of the second molar.

A prerequisite for nonsurgical endodontic treatment of anomalous teeth is a careful clinical and radiographic examination. Many clinicians revealed very complex internal anatomy in double tooth and stressed the significance of knowledge with the root canal morphology before starting the treatment (18-19). During endodontic treatment of a double tooth, the clinician must be prepared for abnormal root canal anatomy and irregular outline of the access cavity. Sometime a multidisciplinary approach to treatment and restoration the function and aesthetic appearance is required. It is important to stress that using higher magnification helped to find and negotiate the root canals more easily in complex cases (19). However, in the present case the canals of supernumerary, mesiobuccal, and distobuccal roots were joined together and positioned like the mesial root of mandibular molars while palatal canal and root position was normal, so the internal anatomy of treated case was very simple.

Successful endodontic treatment depends on careful cleaning, shaping and three-dimensional obturation of the root canal system. Although mechanical debridement of the root canals in double tooth is difficult, but the combination of chemo-mechanical instrumentation and the use of sodium hypochlorite in the present case was sufficient.

## CONCLUSION

In our opinion, an individualized treatment plan is required in double tooth, since these cases may or may not require different treatment modalities compared to normal teeth.
